# Pathology Quality Control for Multiplex Immunofluorescence and Image Analysis Assessment in Longitudinal Studies

**DOI:** 10.3389/fmolb.2021.661222

**Published:** 2021-07-30

**Authors:** Rossana Lazcano, Frank Rojas, Caddie Laberiano, Sharia Hernandez, Edwin Roger Parra

**Affiliations:** Department of Translational Molecular Pathology, The University of Texas MD Anderson Cancer Center, Houston, TX, United States

**Keywords:** digital image analysis, biopsy, quality control, pathology, multiplex immunofluorescence

## Abstract

Immune profiling of formalin-fixed, paraffin-embedded tissues using multiplex immunofluorescence (mIF) staining and image analysis methodology allows for the study of several biomarkers on a single slide. The pathology quality control (PQC) for tumor tissue immune profiling using digital image analysis of core needle biopsies is an important step in any laboratory to avoid wasting time and materials. Although there are currently no established inclusion and exclusion criteria for samples used in this type of assay, a PQC is necessary to achieve accurate and reproducible data. We retrospectively reviewed PQC data from hematoxylin and eosin (H&E) slides and from mIF image analysis samples obtained during 2019. We reviewed a total of 931 reports from core needle biopsy samples; 123 (13.21%) were excluded during the mIF PQC. The most common causes of exclusion were the absence of malignant cells or fewer than 100 malignant cells in the entire section (n = 42, 34.15%), tissue size smaller than 4 × 1 mm (n = 16, 13.01%), fibrotic tissue without inflammatory cells (n = 12, 9.76%), and necrotic tissue (n = 11, 8.94%). Baseline excluded samples had more fibrosis (90 vs 10%) and less necrosis (5 vs 90%) compared with post-treatment excluded samples. The most common excluded organ site of the biopsy was the liver (n = 19, 15.45%), followed by soft tissue (n = 17, 13.82%) and the abdominal region (n = 15, 12.20%). We showed that the PQC is an important step for image analysis and that the absence of malignant cells is the most limiting sample characteristic for mIF image analysis. We also discuss other challenges that pathologists need to consider to report reliable and reproducible image analysis data.

## Introduction

Pathology quality control (PQC) consists of multiple technical steps that evaluate and measure the quality of a sampling process (Adyanthaya and Jose, 2013). PQC also provides consistent checks to identify and address errors and obtain accurate, precise, and reproducible data (Mangino, 2006; Greig, 2019). A retrospective analysis at the National Cancer Institute Developmental Therapeutics Clinic found that 74% of the core needle biopsies performed in pharmacodynamic studies that included fluorescence and mass spectrometry analyses passed their quality control criteria (Ferry-Galow et al., 2016; Parchment and Doroshow, 2016). The study used hematoxylin and eosin (H&E) slide-based analyses as the first PQC step and found that the lack of malignant cells (MCs) excluded the largest number of samples.

In the last 5 years, the immune profiling of formalin-fixed, paraffin-embedded (FFPE) tissues using multiplex immunofluorescence (mIF) staining and digital image analysis methodologies has arisen as a new technology to study several biomarkers on a single slide in longitudinal studies (Francisco-Cruz et al., 2020). However, an efficient PQC process developed by pathologists with experience in digital image analysis is needed. This type of PQC for image analysis and mIF is necessary to avoid expending unnecessary resources and laboratory personnel time (Parra et al., 2020) and to obtain high-quality and reproducible results.

The success of any research study that uses FFPE tissues depends on the quality of the samples. Therefore, it is important to establish minimum parameters for biopsy sample quality that should be met before the staining process begins (Ferry-Galow et al., 2018). Core needle biopsy samples are generally around 1.58 mm in diameter and 12.7 mm long, although their size can vary. The small size of these samples makes them the most challenging for digital image analysis because it is more likely for a significant proportion of the sample to be damaged during cutting, staining, and scanning, especially when sensitive staining methodologies such as mIF are used. Yet, these tissues are invaluable material for longitudinal studies, so efforts to obtain quality data, which is important for translational studies, should be maximized.

The goal of this manuscript is to maximize the workflow of the PQC for digital image analysis. Thus, we retrospectively studied this assessment to standardize the process, to minimize time and cost expenditures, and to guarantee high-quality and reproducible results using mIF and digital image analysis.

## Materials and Methods

From 4,371 biopsies collected by the Adaptive patient-oriented longitudinal learning and optimization program from different research programs at The University of Texas MD Anderson Cancer Center from January through December of 2019, we retrospectively reviewed the PQC reports based on the H&E slides of 931 core needle biopsies from longitudinal studies. Biopsies from different time points were included in this study (608 baseline biopsies and 323 post-treatment biopsies), and all the samples had been processed for mIF and digital image analysis to study the tumor microenvironment, including the presence of cytokeratins, SOX10, and GFAP to characterize malignant cells in different organs; immune checkpoint markers (i.e., PD-L1, B7-H3, B7-H4, IDO-1, VISTA, LAG3, ICOS, TIM3, and OX40); tumor-infiltrating lymphocyte markers (i.e., CD3, CD8, CD45RO, granzyme B, PD-1, and FOXP3); and markers to characterize myeloid-derived suppressor cells (i.e., CD68, CD66b, CD14, CD33, Arg-1, and CD11b), and these samples were placed in panels similar to those previously published (Parra et al., 2021).

Five principal characteristics as annotated in the H&E PQC reports of the biopsies were analyzed: 1) tissue size (length and width), 2) percentage of tumor area with respect to the total size of the sample, 3) percentage of MCs in the tumor area of the sample, 4) percentage of necrotic area, and 5) percentage of fibrosis. In parallel, the PQC of the digital image analysis was retrieved from the final data reports of the mIF panels and reviewed. Similar characteristics were analyzed on the mIF slides. For the cases in which image analysis could not be performed, the comments containing the criterion of exclusion were retrieved instead. All the data from the H&E and digital image analysis PQCs were tabulated, and the results are shown below.

## Results

**FIGURE 1:**
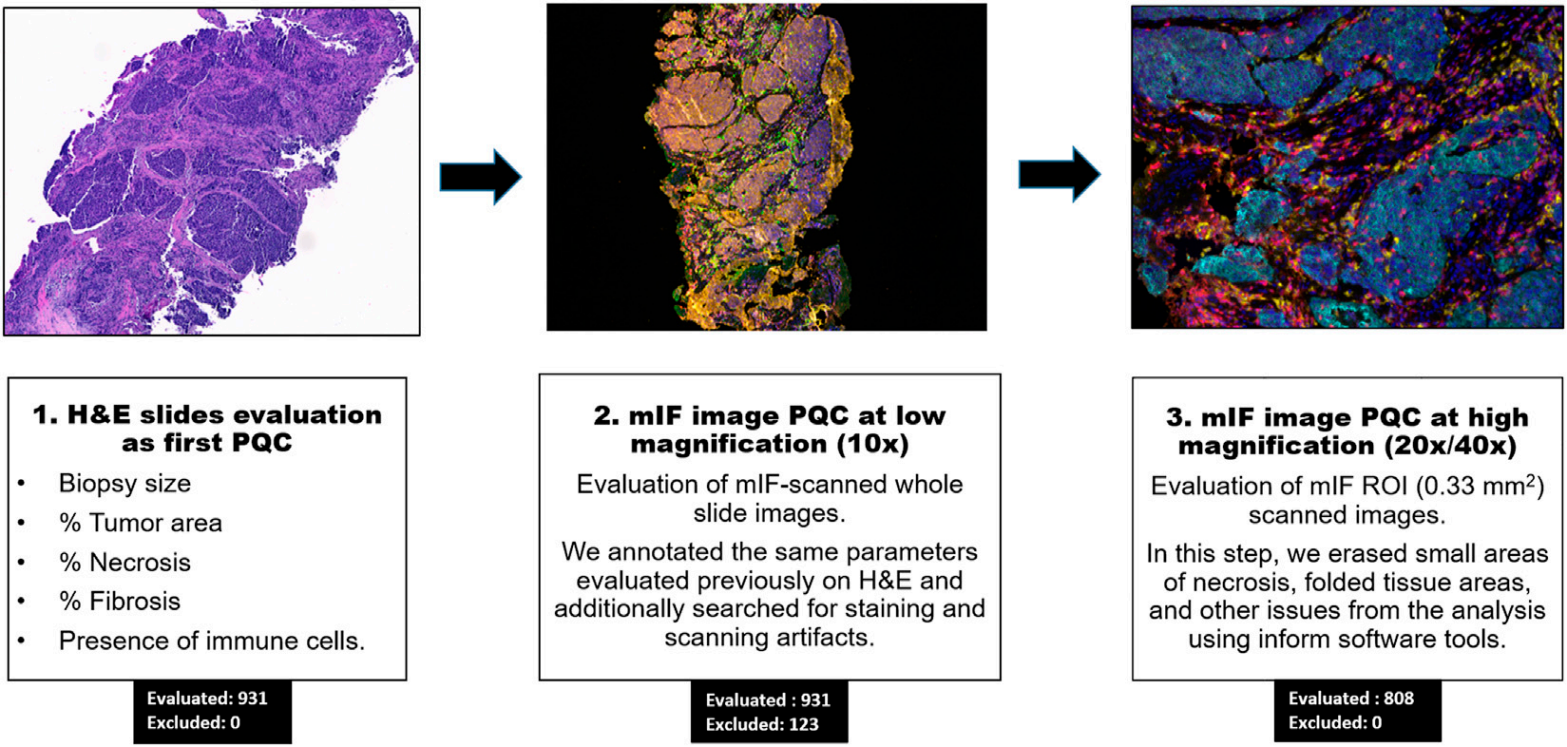
Workflow of pathology quality control (PQC) for core needle biopsy sample assessment for multiplex immunofluorescence and digital image analysis. Showing the overall three steps of PQC, including assessment of the hematoxylin and eosin (H&E) slides for PQC, image analysis PQC at 10x magnification, and image analysis PQC of region of interest (ROI) images at 20x/40x magnification. The numbers of cases evaluated and excluded at each step are indicated.

**FIGURE 2:**
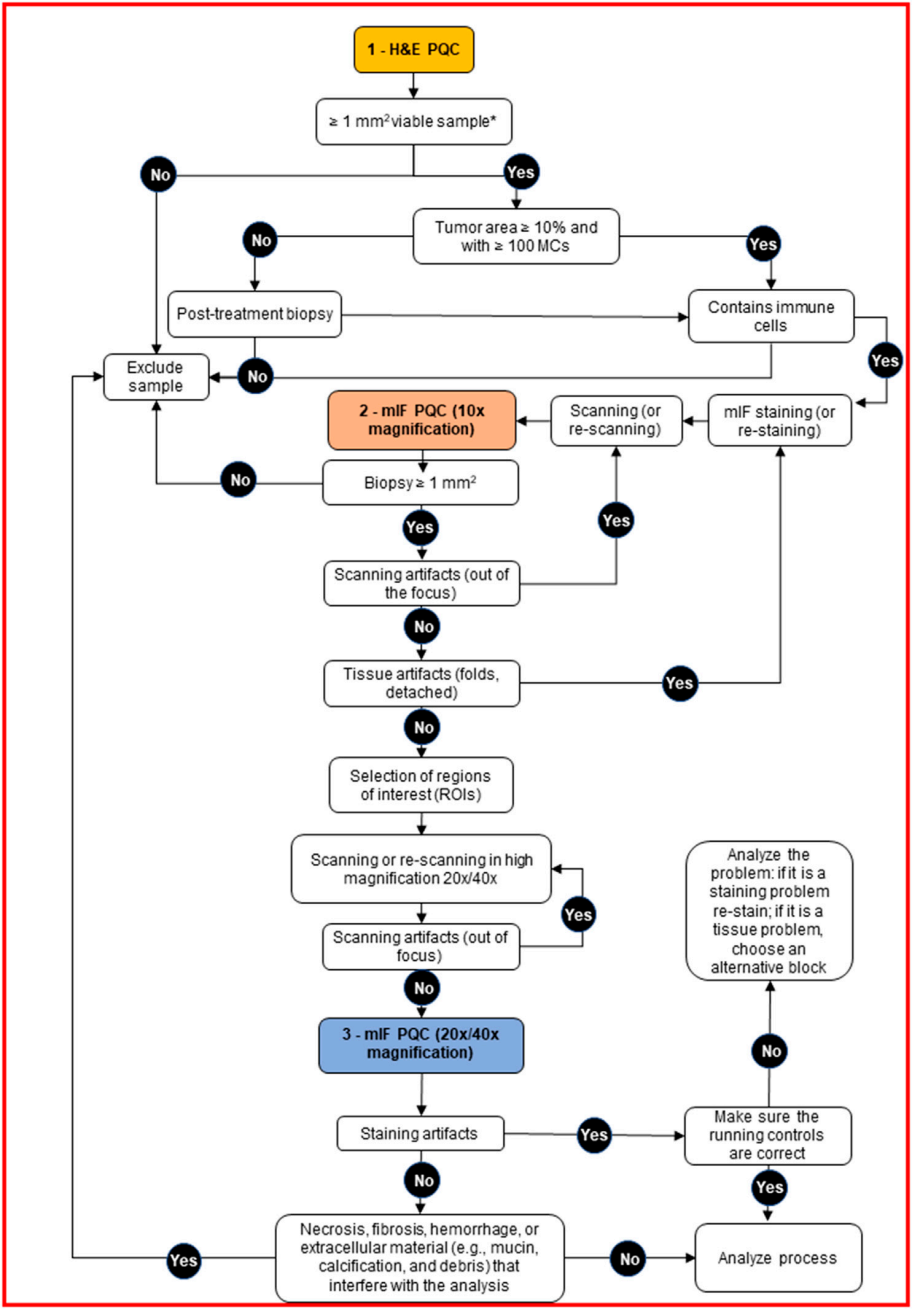
Decision tree for pathology quality control (PQC) of core needle biopsy sample assessment for multiplex immunofluorescence and digital image analysis. The tree shows the detailed protocol with corresponding decisions for the pathologist to make during the three PQC steps. H&E, hematoxylin and eosin; MCs, malignant cells; mIF, multiplex immunofluorescence.

None of the 931 core needle biopsies evaluated were excluded during the H&E PQC, while 123 biopsies (13.21%) were excluded during the digital image analysis PQC at low magnification (10x) (Figures 1, 2). The range of excluded samples per project was 3.45–24.17%. Post-treatment samples were more frequently excluded (62 of 323, 19.20%) compared to the baseline samples (61 of 608, 10.03%). An important characteristic of the samples was their size. The median length was 12 mm (range, 1–24 mm), and the median width was around 1 mm (range, 0.8–1.2 mm). However, we observed that the median length of the samples excluded due to small size was 1.25 mm (range, 0.5–4 mm), and the median width was similar for included and excluded samples.

**FIGURE 3:**
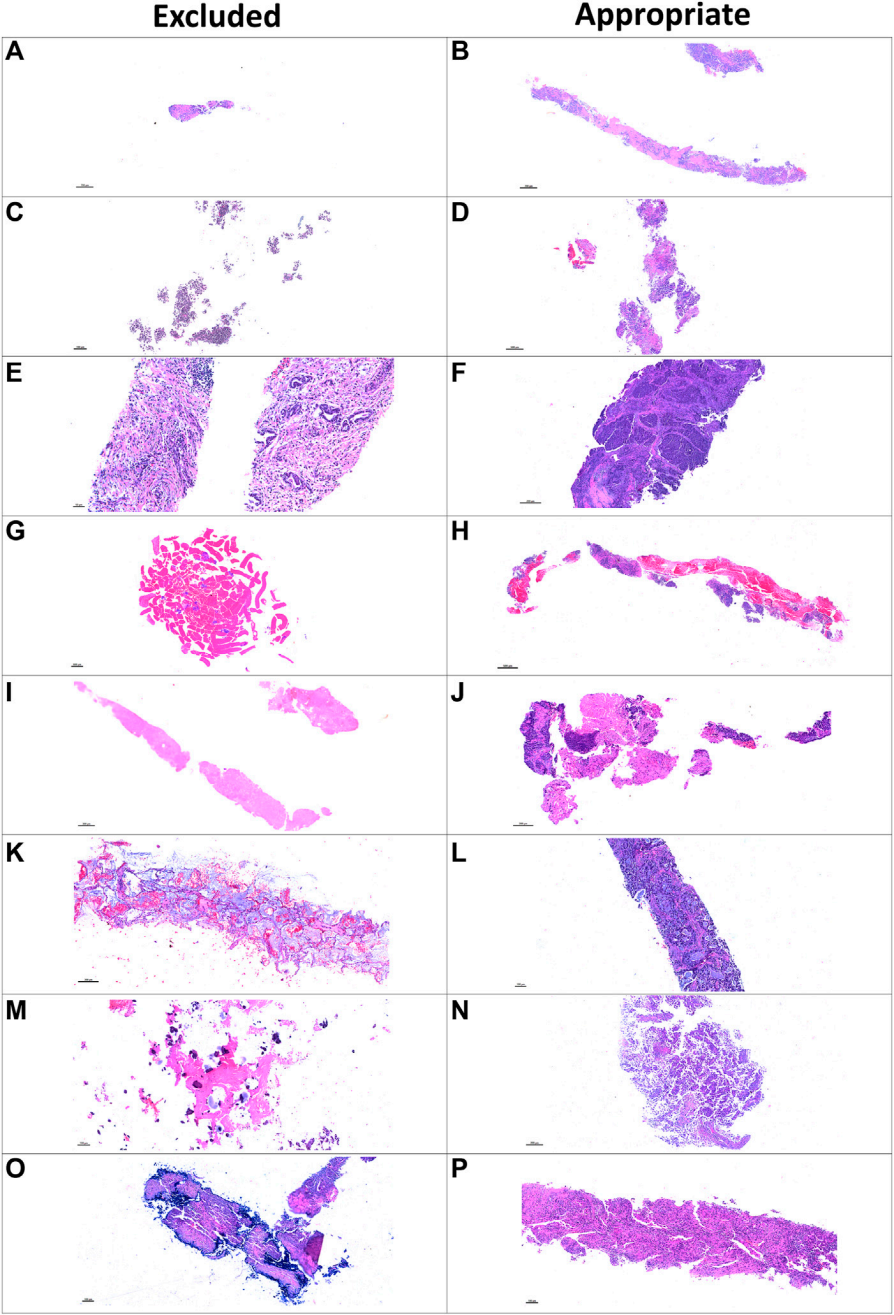
Microphotographs of representative examples of excluded and included core needle biopsies using hematoxylin and eosin slides for pathology quality control. Excluded examples (*left column*) compared with samples considered appropriate for image analysis (*right column*). Small sample size **(A)** compared with a large, adequate sample **(B)**. Sample without malignant cells and only with normal tissue **(C)** compared with a sample with adequate amount of malignant cells **(D)**. Sample with extensive fragmentation **(E)** compared with another fragmented sample that could be included in the analysis **(F)**. Small sample with extensive hemorrhagic area **(G)** compared with another large sample with extensive hemorrhagic area but also with enough tumor content **(H)**. Sample with extensive necrotic area **(I)** compared with another similarly sized sample with enough tumor content for analysis **(J)**. Sample with mucinous and scattered malignant cells **(K)** compared with a sample considered appropriate for image analysis **(L)**. Sample with predominant fibrosis and calcification **(M)** compared with an adequate tumor-containing sample **(N)**. A biopsy with artifact of desiccation **(O)** compared with a well-preserved biopsy **(P)**.

After we retrieved the annotated characteristics of the samples from the H&E PQC reports, we compared the baseline and post-treatment characteristics of the excluded and included samples. (See examples on Figure 3). In the excluded baseline biopsies, the median percentages of tumor area and MCs in the tumor area were both 0% (range, 0–60%). For the included baseline biopsies, the median tumor content area was 95% (range, 30–100%), and the median percentage of MCs in the tumor area was 60% (range, 5–100%). Interestingly, we observed that the excluded baseline samples had tumor areas with a median of 90% fibrotic areas compared to only 20% fibrotic areas in the included baseline samples. The percentage of necrosis was similar in the excluded and included samples. Furthermore, in the excluded post-treatment biopsies, the median tumor area was 10% and the percentage of MCs in the tumor area was 5%. In the included post-treatment samples, the median tumor area was 20% and the percentage of MCs in the tumor area was 50%. We also found a higher percentage of necrotic area in the excluded samples than in the included samples (median, 90 versus 25%, respectively). However, the percentage of fibrotic area was lower in the excluded post-treatment biopsies as shown in (Table 1).

**TABLE 1:** General overview of pathology quality control characteristics in our cohort (*N* = 931) divided by baseline (*N* = 608) and post-treatment (*N* = 323) core needle biopsies.

Biopsy timepoint	Status	N	Characteristic of the sample, median percentage			
			Tumor area	Malignant cells	Fibrosis	Necrosis
Baseline	Included	547	95	60	20	10
	Excluded	61	0	0	90	5
Post-treatment	Included	261	20	50	25	25
	Excluded	62	10	5	10	90

**FIGURE 4:**
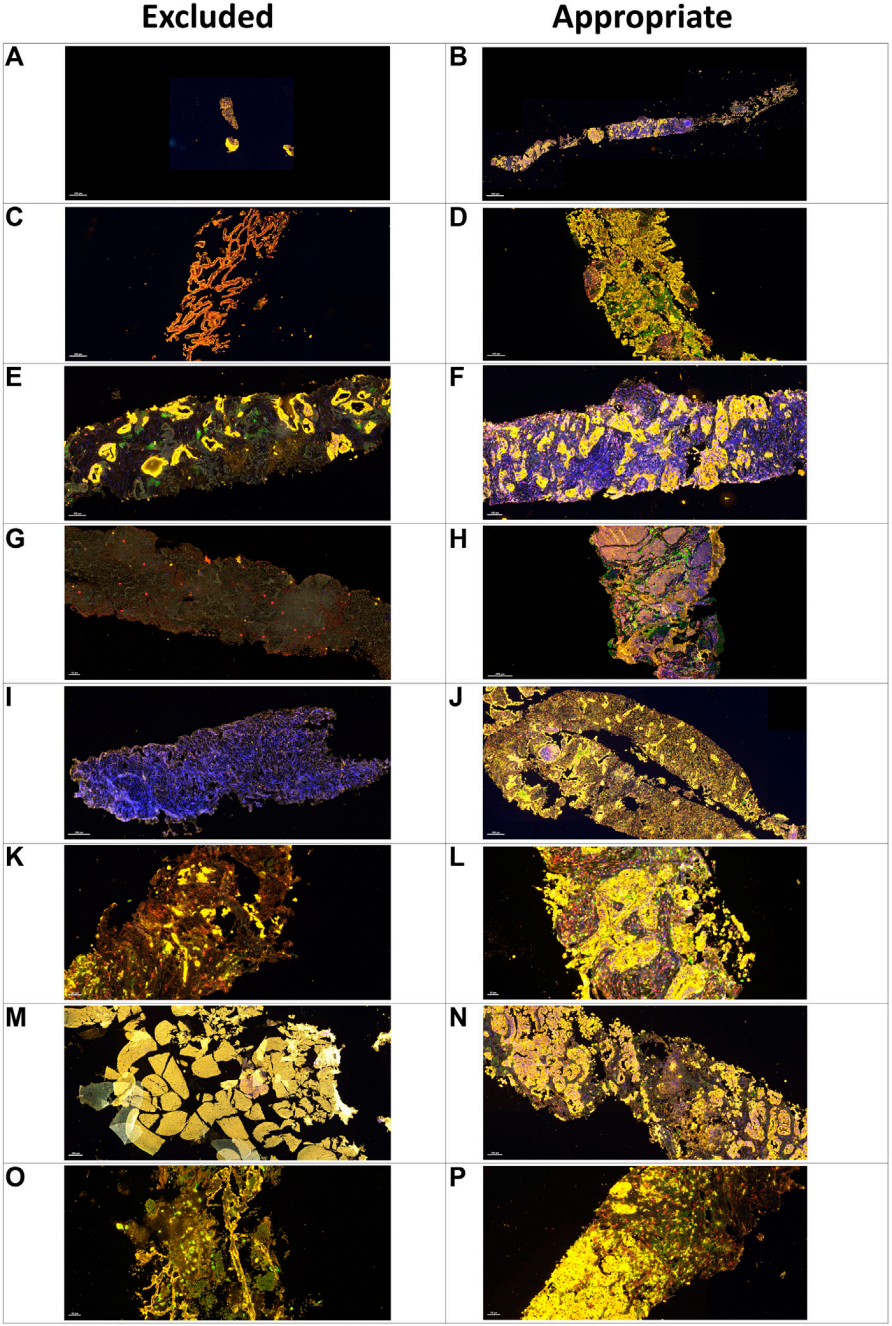
Microphotographs of representative examples of excluded and included core needle biopsies in multiplex immunofluorescence slides using digital image analysis assessment for pathology quality control. Excluded examples (*Left column*) compared with samples considered appropriate for image analysis (*right column*). A small sample **(A)** compared with a large sample with adequate amount of tumor content in *yellow* **(B)**. Sample without malignant cells and with only normal tissue in *yellow* **(C)** compared with nets of malignant cells in *yellow* **(D)**. Nets of malignant cells in *yellow* in the middle of extensive fibrotic areas with lack of inflammatory cells **(E)** compared with a sample with a large amount of inflammatory cells **(F)**. Sample with extensive necrotic area in *grayish green* **(G)** compared with a sample without necrotic areas **(H)**. Sample with staining artifact showing the lack of marker expression **(I)** compared with another sample with adequate staining **(J)**. Sample with crushed cells artifact **(K)** compared with a sample with clear individualization of the different cells **(L)**. A hemorrhagic sample **(M)** compared with a sample with adequate tumor tissue **(N)**. A sample with mostly mucinous material and few tumor cells **(O)** compared with another sample with a regular amount of tumor cells **(P)**.

When we reviewed the digital image analysis PQC reports for the excluded samples, the most common causes of exclusion were absence of MCs or fewer than 100 MCs (n = 42, 34.15%), small tissue sample size (n = 16, 13.01%), mostly fibrotic tissue without inflammatory cells (n = 12, 9.76%), and mostly necrotic tissue (n = 11, 8.94%). The less common reasons for exclusion were fragmentation conditions (n = 2, 1.63%); crushed cell artifact (n = 2, 1.63%); staining artifact, apparently for oxidation and desiccation of the sample (n = 2, 1.63%) and hemorrhagic tissue (n = 1, 0.81%); (Table 2 and Figure 4). Although most of the samples showed one of the previously mentioned predominant causes for exclusion, some samples showed more than one cause for exclusion. For these samples, the most frequent combinatory factors were few or no MCs and mostly fibrotic tissue without inflammatory cells (n = 7, 5.69%) as well as mostly necrotic and fibrotic tissue without inflammatory cells (n = 4, 3.25%) (Table 2).

**TABLE 2:** Characteristics of exclusion criteria observed during digital image analysis PQC (N = 123).

One exclusion criterion	Extent	N (%)
No or fewer than 100 MCs	Entire sample	42 (34.15)
Small biopsy size (< 1 mm^2^)	Entire sample	16 (13.01)
Tissue availability after staining	Entire sample	14 (11.38)
Fibrotic tissue without inflammatory cells	More than 80%	12 (9.76)
Necrotic tissue	More than 80%	11 (8.94)
Fragmented biopsy	Entire sample	2 (1.63)
Staining artifact of oxidation/desiccation	Entire sample	2 (1.63)
Crushed cells artifact	Entire sample	2 (1.63)
Mostly hemorrhagic tissue	Entire sample	1 (0.81)
**Two exclusion criteria**		
No MCs or fewer than 100 MCs and fibrotic tissue without inflammatory cells	More than 80%	7 (5.69)
Necrotic tissue and fibrotic tissue without inflammatory cells	Entire sample	4 (3.25)
Fragmented biopsy and staining artifact of oxidation/desiccation	Entire sample	3 (2.44)
Small biopsy size and necrotic tissue	More than 80%	2 (1.63)
Necrotic tissue and crushed cells artifact	Entire sample	2 (1.63)
Small biopsy size and fibrotic tissue without inflammatory cells	More than 80%	1 (0.81)
No MCs or fewer than 100 MCs and necrotic tissue	More than 80%	1 (0.81)
Staining artifact of oxidation/desiccation and crushed cells artifact	Entire tissue	1 (0.81)
**Total**	**123**	**123 (100)**

PQC, pathology quality control; MC, malignant cell.

**FIGURE 5:**
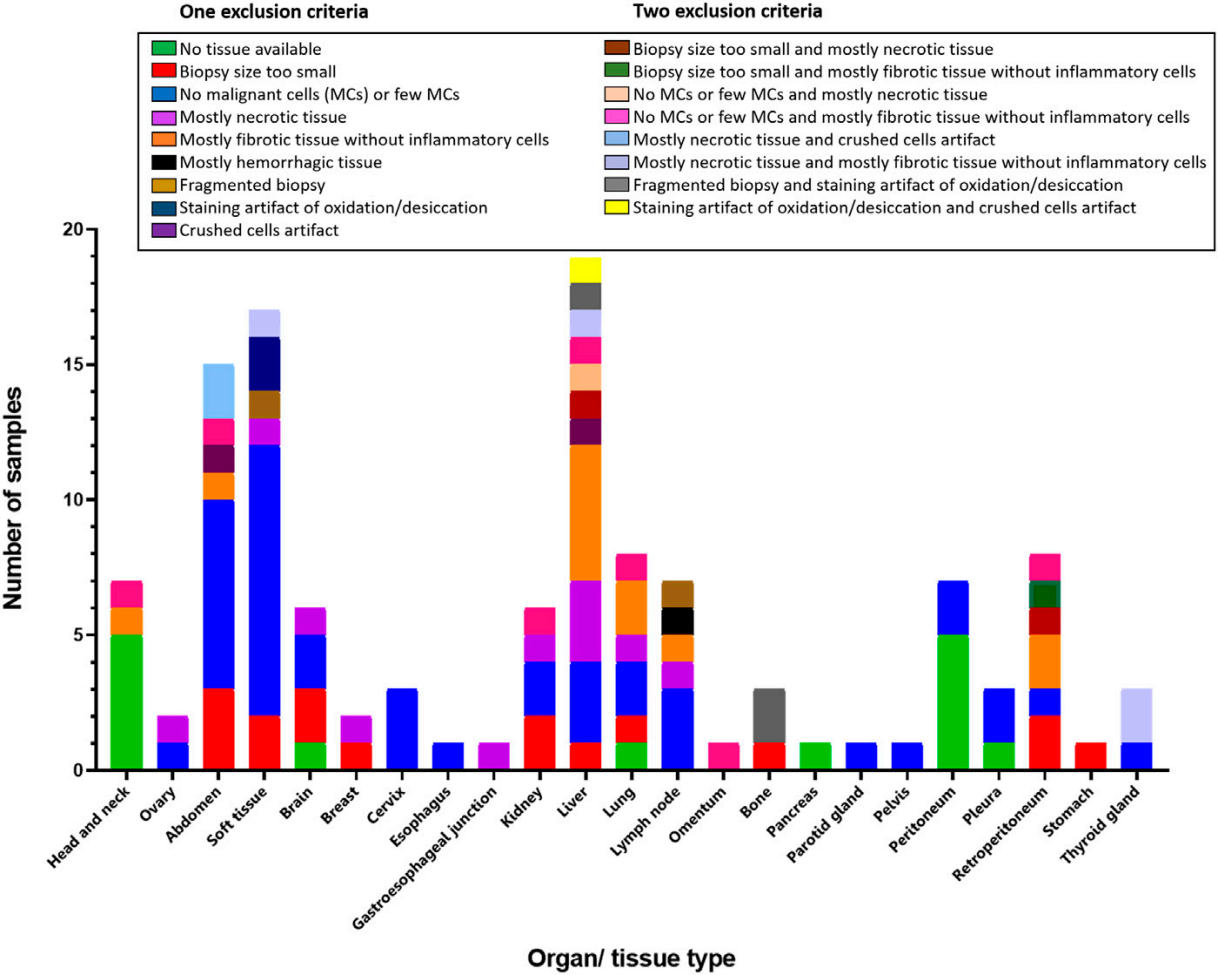
Bar graph showing localization and exclusion criteria of the samples. Inset box containing the exclusion criteria divided in one or two criteria.

With respect to the site of the biopsy, the liver had the most samples excluded (19 of 123, 15.45%), followed by soft tissues (17 of 123, 13.82%) and the abdominal region (15 of 123, 12.20%). The remaining excluded samples came from a wide range of anatomic locations, such as the breast, cervix, gastrointestinal tract, lung, and lymph node, and none of these sites alone accounted for more than 10% of the total excluded samples (Table 3). It was possible to identify differences in the causes of exclusion in the context of the biopsy location. For example, liver biopsies were excluded more frequently due to fibrotic areas without inflammation, whereas soft tissue samples were excluded more frequently for having few or no MCs (Figure 5).

**TABLE 3:** Location of excluded core needle biopsies.

Location	N (%)
Connective tissue, head and neck	7 (5.69)
Ovary	2 (1.63)
Abdomen	15 (12.20)
Soft tissue	17 (13.82)
Brain	6 (4.88)
Breast	2 (1.63)
Cervix	3 (2.44)
Esophagus	1 (0.81)
Gastroesophageal junction	1 (0.81)
Kidney	6 (4.88)
Liver	19 (15.45)
Lung	8 (6.50)
Lymph node	7 (5.69)
Omentum	1 (0.81)
Bone	2 (1.63)
Pancreas	1 (0.81)
Parotid gland	1 (0.81)
Pelvis	1 (0.81)
Peritoneum	7 (5.69)
Pleura	3 (2.44)
Retroperitoneum	8 (6.50)
Sternum	1 (0.81)
Stomach	1 (0.81)
Thyroid gland	3 (2.44)
Total	123 (100)

## Discussion

This study shows that different characteristics of core needle biopsies can impede digital image analysis, and PQC specific to digital image analysis can help guarantee high-quality and reproducible data. In this study, we observed that sample size, tumor content, percentage of necrosis, and percentage of fibrosis are important in quality control of physical and scanned H&E slides as well as scanned mIF slides. We also showed that a systematic PQC assessment of core needle biopsies is important to maintain the quality of the biopsies for image analysis.

According to our study, tissue size and tumor content were the most challenging and important characteristics for determining which samples could undergo digital image analysis to study the phenotypes expressed by the tumor immune microenvironment and MCs. We showed that 34.15% of the samples were excluded owing to the absence of MCs or low tumor content, and 13.01% of the samples were excluded owing to small sample size. These excluded samples had a median size of 1.25 x 1 mm. Similar to a previous study in which the most important exclusion criteria was the absence of MCs, 44% of the biopsy specimens evaluated in this study contained less than 25% viable MCs (Pisano et al., 2001). As we expected, in these core needle biopsy samples the most important measure that differentiated excluded and included biopsies was sample length, given that sample widths were determined by the different needle diameters as well as the fixation process.

As previously published (Parra et al., 2020), we noted that a tumor content of at least 10% in a biopsy sample that is at least 2 × 1 mm is enough to perform image analysis; however, we can successfully stain samples as small as 0.5 mm^2^. The idea that these samples are representative of the entire tumor microenvironment is still controversial due to intratumoral heterogeneity in biomarker expression (Nicoś et al., 2020). Thus, we recommend an area of analysis at least 1 mm^2^ to obtain reliable data from this type of sample, but this minimum area will vary depending on the tumor content of the sample (Padmanabhan et al., 2017). For example, in the literature there are publications that considered samples 10 mm in length to be adequate for the diagnosis of prostate cancer (Cicione et al., 2012) and 15 mm in length adequate for the diagnosis of liver disease (Palmer et al., 2014). Another study using mIF on pre-treatment biopsies and post-treatment tumor resections of breast carcinoma found that adequate tissue sampling, with at least 15 regions of interest, was necessary to have a strong correlation between the tumor-infiltrating lymphocytes and PD-L1 markers included in an mIF panel and the H&E/PD-L1 clone SP142 clinical assays (Sanchez et al., 2021). However, there are not standardized image analysis PQC protocols to determine the minimum sample size needed for immunoprofiling, and more studies are warranted to address this need. We believe that each sample should be evaluated separately, according to its type (whole section or core needle biopsy) and the study aims.

When comparing baseline and post-treatment biopsies, the median tumor content was 90 vs 20%, respectively. While an adequate tumor presence is required in baseline biopsy samples, fewer MCs or an absence of tumor cells in cases with complete pathological response after treatment is appropriate in post-treatment biopsy samples. Regarding the minimum number of MCs needed to analyze specific marker clones that are expressed by MCs, such as PD-L1, at least 100 MCs are recommended to obtain consistent and reliable data (Tsao et al., 2018; Francisco-Cruz et al., 2020). However, there is not a consensus regarding the minimum number of MCs needed for image analysis of different markers expressed by MCs. According to our experience, we believe that a minimum of 100 MCs is needed to consider a sample as representative for digital image analysis. If the sample has fewer than 100 MCs, then we consider it to be inadequate to perform image analysis to study markers expressed by those cells. It is important to consider that when performing immune profiling for longitudinal studies, we work not only with baseline biopsies that need to contain enough MCs but also with post-treatment biopsies that many times lack enough MCs because of the effects of treatment. In these cases and when the study is not related to a specific marker expressed by MCs, exclusion of the sample shoud also be based on criteria other than the number of MCs, including the proportion of inflammatory cells, especially T-cells that play an important role in the tumor immune response, and other components such as fibrosis, edema, or necrosis (Hellmann et al., 2014).

The presence of inflammation in the tumor and stroma compartment is required when the aim of the study is to quantify the immune microenvironment (Parra et al., 2020). However, there is a lack of consensus on the areas that adequately show the inflammatory microenvironment. Thus, the pathologist must subjectively define an adequate area. After the mIF slides are scanned, the pathologist should always try to select the entire tumor area in the sample. However, they must capture at least 1 mm^2^ of the regions of interest (Parra et al., 2020) to obtain reliable data

In our daily routine, we always look for characteristics such as inflammatory cells forming aggregates, as tertiary lymphoid structures (Sautès-Fridman et al., 2019) or in a diffuse distribution, as these can direct the analysis toward a reliable minimum quantity of cell phenotypes to obtain comprehensive data to be correlated with the clinicopathologic component. However, there is not a universal minimum number of cell phenotypes considered to be an adequate representation of the sample, partly because this number depends on the biological characteristics of the tumor and because we are often limited by software, which requires a minimum of five cells expressing a marker per sample to start the image analysis process.

Fibrotic samples without inflammatory cells are another important cause of exclusion. In our cohort, we excluded 9.76% of our samples because of this criterion in both baseline and post-treatment samples. However, we found that our baseline and post-treatment samples had similar fibrotic content (20–25%). Curiously, we found that excluded baseline samples had more fibrotic content than excluded post-treatment samples (90 versus 10%, respectively). As expected, one of the important exclusion factors for post-treatment samples was necrosis, which was often a result of the treatments’ effects on tumors. Although this characteristic is often evaluated as a positive sign of treatment response, it is a limiting factor for digital image analysis (Parra et al., 2020).

Tissue artifact-related sample exclusion was less frequent (1.63%). When the H&E PQC is performed properly, these tissue artifacts could be related to the effects of surgical trauma, tissue ischemia, poor fixation, cutting procedures, or scanning problems (Flamminio et al., 2011). Even subtle artifacts can have large implications for the algorithms used to recognize positive biomarkers, resulting in inaccuracies. For this reason, there have been many attempts to create digital pathology tools for automated PQC (Ameisen et al., 2013; Senaras et al., 2018; Bengtsson and Ranefall, 2019). Software for automated PQC that employs image metrics and identifies H&E scanned slides with gross technical artifacts exists, but it is not suitable for use on mIF-stained slides (Janowczyk et al., 2019).

We also observed that some specific organ site characteristics can interfere with the image analysis and thus are extremely important in digital imaging analysis PQC. The most often excluded biopsy site was the liver, with extensive fibrosis as the most common exclusion criterion. Soft tissue samples were excluded the second most often, mainly for absence of or few MCs. Nevertheless, these high rates of exclusion could be related to the high numbers of liver and soft tissue projects included in our study. Each location or organ has its own technical specifications for obtaining an adequate sample. For example, for breast cancers, some authors have described that the use of the semi-automated needle yielded a 23% rate of inadequate results compared to 9% when using an automated needle to obtain breast samples (Sridharan et al., 2015). Different needle sizes are recommended depending on the organ and its vascularization status to avoid the risk of hemorrhage, especially in liver samples (Hall et al., 2017; Hoang et al., 2018). However, the use of different needle sizes did not to affect the quality of the biopsy of breast tissue (Huang et al., 2017). For these reasons, each specialist must analyze the risks and benefits of the selected biopsy technique and its effect on the quality of samples.

Finally, other tissue characteristics that should be avoided for the mIF analysis but were not found to be exclusion criteria in the current study are the presence of noncellular materials, e.g., glandular secretions; intra-alveolar material, which may contain inflammatory cells and debris; cartilage; bone tissue, in which decalcification may affect tissue staining; and adipose tissue, which can lead to tissue detachment during the staining process (Parra et al., 2021).

In conclusion, PQC for digital image analysis for mIF is extremely important to obtain reliable results. However, consensus and guidelines are necessary to produce reliable data in multi-institutional longitudinal studies. Evaluation of H&E slides at the beginning of any process as well as evaluation of mIF image slides for digital image analysis is fundamental and should consider the study design and material received, including the markers included in the mIF panels.

## Data Availability

The raw data supporting the conclusion of this article will be made available by the authors, without undue reservation.
